# Clinic of Prof. Davis in the Mercy Hospital

**Published:** 1861-03

**Authors:** F. S. C. Grayston

**Affiliations:** Member of Senior Class Med. Department of Lind University


### CLINIC OF Prof. DAVIS IN THE MERCY HOSPITAL.

Reported by F. S. C. Grayston, Member of Senior Class Med. Department of Lind University.

*Diphtheria.*—On entering the ward, Prof. Davis remarked, in substance, as follows:

The patient before you, gentlemen, is a little boy, aged about three years. He has been sick two days. You see his face slightly flushed; his lips look dry; his skin is dry and moderately hot; his pulse 110 per minute, but soft; you see a fullness or swelling behind the angle of the jaw on both sides, and a little down the neck, which, by the touch, you find occasioned by enlargement and induration of the tonsils, and some of the lymphatic glands; and on opening the mouth, you see the tongue covered with a white fur, the tonsils much swollen, and covered with particles of a white membranous exudation, and the mucous membrane of the whole interior fauces red and swollen.

The breath is moderately offensive; the nostrils partially obstructed by swelling of the schneiderian membrane; deglutition is somewhat difficult and painful; the urine scanty; and the fecal evacuations nearly normal. These symptoms indicate a case of Diphtheria, of about the average degree of severity. You readily recognize the close resemblance of this case to one your attention was called to, briefly, two or three days since, and which still occupies the adjoining bed. In that case, the fever was, at first, more active, the pulse more full, and the face more flushed. But there was the same swelling of the tonsils and glands behind the angle of the jaw, the same inflammation of the mucous membrane of the fauces, with a white membranous exudation over the greater part of the surface, which serve to distinguish Diphtheria, from ordinary inflammation of the tonsils and mucous membrane of the fauces.

Many have supposed that Diphtheria was a new form of disease, with which the profession was entirely unacquainted until within the last four or five years. This error, doubtless originated from the general adoption of a new name.

Until a very recent period, nearly all the inflammatory affections of the fauces, pharynx, larynx and trachea, were described under the general name *angina.* Hence, the terms catarrhal angina, suffocative angina, putrid angina, &c., are familiar to those who peruse the medical writings of half, or even of a quarter of a century since. Under some of these designations, Dr. Samuel Bard, and others, more than half a century since, described the present disease with almost as much minuteness and accuracy as any of the writers of the present day.

The word *Diphtheria*, was first used by certain French writers to designate all that class of anginose affections, that were accompanied by a fibrous or membranous exudation upon the surface of the mucous membrane. From this, the word Diphtheria has been applied particularly to the disease before us, and passed fully into popular use.

*Causes.*—In relation to the causes of Diphtheria, the lecturer stated that we knew but very little. It has prevailed at all seasons of the year; on almost every variety of soil and geological formation; in populous cities and rural districts; and in every degree of severity, from the mildest sporadic cases to the most malignant and fatal epidemic. To say that it originates from some epidemic condition of the atmosphere, is only another mode of confessing our ignorance; for we know nothing concerning the nature of such *epidemic* condition. Many regard the disease as contagious, and consequently produced by a specific poison. Without pretending either to affirm or deny the existence of contagiousness, we must say, that although the disease has been moderately prevalent in this city for eighteen months past, yet we have not seen any adequate evidence of its propagation by contagion. True, we have seen two, three, and four children attacked with it in quick succession in the same family; but in very many other families we have seen a single child attacked, in the midst of three or four others, all of whom escaped without a symptom of the disease. The very child before us, is from the adjoining orphan asylum where 40 or 50 children were fully exposed to the disease more than two weeks since, and yet up to the present time only three have been attacked.

*Symptoms.*—The lecturer remarked that the symptoms already pointed out in the patient before us, very correctly represented the great majority of cases of Diphtheria in the early part of their progress. In some, however, the symptoms are more mild, there being very little febrile excitement, no swelling of the lymphatic glands, and only a superficial inflammation of the fauces with a few spots of diphtheritic exudation. On the other hand, some cases occur, in which the febrile symptoms are severe; the glands of the neck and throat swell rapidly; while the surface of the mucous membrane of the fauces, pharynx, &c., become so rapidly covered with a thick, tenacious, layer of false membrane, that suffocation may ensue in a few hours. In the great majority of cases, there is decided febrile action. The pulse, however, is rarely *firm* under the finger, it is for the most part, quick, sharp, and *compressible*; the skin is dry, the urine scanty, (sometimes none for 24 hours,) with little or no thirst; while a remarkable degree of prostration is noticed early in the progress of the disease. Still in this particular, (as in all others,) there is considerable difference, some cases present throughout their entire course all the symptoms of a *stenic* disease; whilst the great majority, (eight out of every ten) present the typhoid form of symptoms, the pulse being *soft* and *yielding*, countenance pale, respiration slow, and mental faculties dull. The local symptoms, though varying much in intensity, show certain tendencies with much uniformity. In almost all instances, after the first three or four days, the breath is offensive, the saliva abundant, and a thin sanious discharge takes place from the nostrils. A little later, the membranous exudation in the fauces will be found breaking up and disappearing in matter expectorated, the latter being muco-purulent with shreds of white fibrous matter. In cases progressing favorably, the redness and tumefaction of parts within the fauces diminish, deglutition is easier, the breathing less noisy, and the external glandular swellings slowly disappear. In less favorable cases, as the membranous exudation becomes detached, extensive superficial ulcerations are found in the fauces, keeping up an abundant and offensive muco-purulent secretion; while the same supurative process extends to the schneiderian membrane, causing the nostrils to be obstructed with the same kind of secretion.

In cases still more severe, instead of superficial ulcerations, deep gangrenous sloughs form, in the tonsils and different parts of the soft palate; the breath emits a putrid odor; deglutition is nearly or quite suspended; and the general symptoms become profoundly typhoid. When the local inflammation extends into the larynx, we have added, the characteristic cough and breathing of croup. This may take place at the commencement of the disease, or it may supervene at any subsequent period, even after the inflammation in the fauces has began to improve.

*Patholgy.*—In regard to the nature or special Pathology of Diphtheria, we would state briefly, that it is a general disease involving, both an altered condition of the properties of the solids and the composition of the fluids, of the body; with a special tendency to develop certain local inflammations in the course of its progress. That the quality of the blood and the vital affinity of the organized textures throughout the body, are altered, is clearly proved by those cases in which false membrane or fibrinous exudations appear on abraded surfaces in different parts of the body. It is but a few weeks since, that we saw a young man with a diphtheritic membrane firmly adherent to the exterior middle part of the upper lip. Near the same time, a patient from whose axilla a tumor had been extirpated, and the wound had rapidly filled up with healthy granulations, was attacked with the ordinary symptoms of Diphtheria in a mild form, and within twenty-four hours, the whole surface of the wound was covered with a thick layer of false membrane. In two or three days this was broken up and detached, but the healthy granulations had also disappeared, and a thin sero-purulent, offensive discharge continued for several days. These facts with many others of similar nature, to be found on record, show plainly, that the disease is by no means local, but that it involves a Pathological condition of the solids and fluids generally. But what is this pathological condition? From much observation at the bed-side as well as a careful study of what has been written on the subject, I am satisfied that the general pathological condition, consists in such an alteration of vital affinity, or the relation of the blood to the secreting structures, that the latter fail to eliminate the normal amount of effete nitrogenous matter derived from the metromorphosis of the tissues. Hence, fibrinous material rapidly accumulates in the blood and becomes effused in the form of a false membrane, upon every inflamed or abraded surface. This accumulation of effete matter further degenerates the blood, and aids to induce that early tendency to prostration, with irritation and fœtid secretions which have been noticed by almost all writers on the subject. The fœtid breath, the dry skin, the scanty urine, and the large proportion of cases in which the latter has been found albuminous, all corroborate this view. While such is the general pathological condition of the system, there is a special tendency to local inflammation of the fauces, pharynx, larynx, and lymphatic glands of the neck. As might be expected from the condition of the blood, already described, whenever inflammation occurs in a membrane, its surface first becomes coated with a white fibrinous exudation, followed by a disposition to ulceration or gangrene; and the inflamed glands become early infiltrated with the same caco-plastic or fibrinous material, causing swelling and induration, which disappear very slowly during the subsequent convalesence.

*Prognosis.*—The mortality from Diphtheria varies much in different seasons and different localities. In this respect, it bears a close analogy to Scarlatina.

Some cases are so mild as to require no other care than proper hygienic regulations, while others are so severe as to reach a fatal result in two or three days. As the disease has prevailed in this city during the last eighteen months, and directly within the circle of my own practice, the ratio of mortality has been *one* in twenty-five. We think there are three methods by which Diphtheria may reach a fatal result. The first, appears to consist in so profound an alteration of the blood and properties of the solids, that the capillary circulation rapidly fails; the mind drowsy and lethargic; the urine very scanty; and the inflamed parts of the throat gangrenous. In this class of cases, death often takes place during the first week after the attack.

In the second class of fatal cases, the fever is more active, the sensibilities more acute, while the inflammation in the fauces and glands, is more intense. But when the diphtheritic exudation begins to be thrown off, the parts are found extensively ulcerated. The same process extends to the nasal passages, and sometimes to the ears, causing copious muco purulent and fœtid discharges from all these parts. The glands remain swollen and indurated. The patient rapidly looses flesh and strength; and during the latter part of the second week, diarrhœa supervenes and the patient dies asthenic. The third mode of reaching a fatal result, is by the extension of the local inflammation to the mucous membrane of the larynx, trachea, and sometimes, bronchial tubes.

In some of the cases belonging to this class, inflammation and the formation of the false membrane, occur in the larynx, at the very commencement of the disease, causing at the outset, characteristic cough and breathing of croup. In such suffocation sometimes ensues with twenty-four or forty eight hours. In other cases of this class, the laryngial or croupy symptoms do not occur until from five to ten days after the commencement of the disease. Then the cough suddenly becomes croupy, the voice suppressed, the respiration difficult, and suffocation ensues more from œdematous infiltration into the sub-mucous cellular tissue of the glottis than from the formation of false membrane. Two of the four fatal cases that have occurred in my own practice during the past year, died in this manner.

A third, was the case of an adult lady, in whom the pseudomembranous inflammation early extended to the whole bronchial mucous membrane. A knowledge of these different modes of reaching a fatal result, and a correct appreciation of the tendency of each individual case, is of much practical importance; as it will enable you to so guide your treatment so as to more fully counteract the unfavorable tendencies, and thereby conduct the case to a favorable termination.

*Treatment.*—The lecturer remarked, that so large a part of the clinic hour had passed, that he would not even allude to the various methods of treatment, which had been recommended by writers and practitioners, but would detail, as briefly as possible, his own views on the subject. From the views already presented concerning the pathology of the disease, we may deduce three rational indications for treatment. First, to preserve the properties of the blood and tissues from degeneration or impairment. Second, to promote elimination, especially from the skin and kidneys. Third, to relieve the local inflammation in the throat and adjacent parts. For fulfilling the first indication, we are in the habit of relying on the use of the chlorates of Potassa and Soda, Sulphate of Quinine, and Tincture of Chloride of Iron. To meet the second indication, we use Nitrous Ether and Tinc. Verat. Viride, Dover's Powder and Calomel, or Dover's Powder and Camphor, according to the activity of the fever and the stage of the disease. In the case before us, we shall endeavor to meet both these indications by the two following prescriptions, viz :

℞ Chlorate of Potassa, ʒ jj.

Hydrochloric Acid fl. ʒ ss.

Water, ℥ jjj.

Mix, and give a tea-spoonful in half a table-spoonful of sweetened water every four hours.

℞ Sulph. Quinine, 6 grs.
Pulv. Doveri, 6 grs.

Mix, divide into six powders, and give one every four hours, half way between the doses of the Chlorate Solution. To combat the local inflammation, in those cases presenting much tumefaction of the tonsils and other glands of the neck, we direct for the first twenty-four hours, the constant application of cloths wet in an infusion of Aconite Leaves and Muriate of Ammonia ; after this we have the neck and parotid region wet every three hours with the following liniment, viz :

℞ Ol. Olive, ℥ jj.

Ol. Terebinth, ℥ ss.

Chloroform, ℥ ss.

Mix.

This liniment we shall direct to be used for the little patient before us. We shall make no local application either by sponge or pencil to fauces. Our experience has satisfied us that in all the milder cases, especially in young children, the irritation produced by the application of strong solutions of Nitrate of Silver, Sulphate of Copper, Tincture of Iron, &c., &c., by forcing a sponge into the throat, does more harm than good. Swallowing the acidulated solution of Chlorate of Potassa every two or three hours produces a better effect on the throat than the stronger applications. In the second stage of the more severe cases, when the throat is much obstructed by false membrane or tenacious mucous, we prefer the application once a day of a solution of Iodine, Sulph. Zinc, or Sulph. Copper, made carefully with the finest quality of sponge or a camel hair pencil. It is only in the more decidedly active class of cases, and only during the first two days that we use arterial sedatives or alterative doses of mercurials. If the cases prove protracted, and typhoid symptoms predominate, we rely mostly on a combination of Quinine, Pulv. Doveri and G. Camphor, alternated with Tinc. Ferri Chloridi; and nourishment. We also continue to give the Solution of Chlorate of Potassa or Soda, partly for the local effect on the throat, and also to increase the capacity of the blood for the absorption of oxygen.

In those cases of Diphtheria in which the inflammation attacks the Larynx, indicating the ordinary symptoms of croup, we pursue the same course of treatment in all respects, with the addition of an emetic at suitable intervals. When the Laryngeal or croupy symptoms appear with the commencement of the attack, we loose no time in giving from 3 to 5 grains of the Sub-Sulphate of Mercury, and repeating in thirty minutes if the first does not produce free vomiting. We have met with three or four well marked cases, in which this checked the laryngial symptoms at once, and they subsequently progressed to a favorable termination like ordinary cases of Diphtheria. In some other cases we have been obliged to repeat the emetic doses of the Sub Sulphate of Mercury, in from four to six hours during the whole of the first two days, and yet have reached a favorable result.

When the laryngeal symptoms supervene, after the Diphtheritic disease has existed from five to ten days, there is much more danger of suffocation from infiltration into the sub-mucous cellular tissue, constituting œdema of the glottis, than from false-membrane. In such cases, we have thought the Pulv. Alum, as recommended by Dr. Meigs in croup, the best emetic; while the oxygenation of the blood and the tone of the capillary vessels must be sustained by the constant use of the chlorates and Tinc. Ferri. Chloridi, with suitable nourishment. A few words in regard to the sequalæ of Diphtheria. Impoverishment of the blood; chronic suppurative inflammation in the lining membrane of the nostrils, external ears, and glands of the neck; are observed during convalesence from this disease, much as they are after scarlatina. And they may be treated in the same manner. But there is another affection of a singular nature which has been observed to accompany and follow attacks of Diphtheria. We allude to paralysis of the muscles concerned in deglutition. It is generally first noticed immediately after convalescence, though we have seen some cases in which it evidently existed during all the latter stage of the disease. It is indicated by a relaxed and pendulous condition of the uvula and soft palate; by the latter falling backward and causing snoring and obstructed breathing when the patient sleeps; by awkwardness of swallowing and frequent regurgitation of liquids through the nostrils; and by aphonia or a peculiar nasal tone to the voice. In two cases that have come under our observation, the paralysis was so complete that deglutition was entirely suspended, although the diphtheritic disease had disappeared and there were no traces of inflammation or ulceration left in the fauces or pharynx. These patients were sustained for several days solely by nutritious injections, consisting of milk and beef-tea. The remedy on which we principally rely for the removal of this paralysis, is Strychnine and Iron, as in the following formula, viz:

| | | | |
|---|---|---|---|
| ℞ | Strychnine, | 1 gr. | |
| | Citrate of Iron, | ʒ j. | |
| | Citric Acid, | ʒ j. | |
| | Water. | ℥ jj. | Mix. |

The dose must be suited to the age of the patient, and may vary from ten drops to a tea-spoonful, given in sweetened water. When the patient cannot swallow, we give the same medicine, in twice the above doses, per rectum along with the nutritive enemas. The dose should be repeated in from four to six hours, and the remedy continued until the paralysis is removed. In all such cases, a nutritious diet, and exposure to fresh pure air should be given to the patient to as great an extent as practicable.

